# Wireless monitoring and real-time adaptive predictive indicator of deterioration

**DOI:** 10.1038/s41598-020-67835-4

**Published:** 2020-07-09

**Authors:** Heather P. Duncan, Balazs Fule, Iain Rice, Alice J. Sitch, David Lowe

**Affiliations:** 1 Birmingham Children’s Hospital, Steelhouse Lane, B4 6NH UK; 2 Aston University, Birmingham, UK; 3 Institute of Applied Health Research, University of Birmingham, Birmingham, UK

**Keywords:** Predictive markers, Prognostic markers, Paediatric research, Biomarkers, Medical research

## Abstract

To assist in the early warning of deterioration in hospitalised children we studied the feasibility of collecting continuous wireless physiological data using Lifetouch (ECG-derived heart and respiratory rate) and WristOx2 (pulse-oximetry and derived pulse rate) sensors. We compared our bedside paediatric early warning (PEW) score and a machine learning automated approach: a Real-time Adaptive Predictive Indicator of Deterioration (RAPID) to identify children experiencing significant clinical deterioration. 982 patients contributed 7,073,486 min during 1,263 monitoring sessions. The proportion of intended monitoring time was 93% for Lifetouch and 55% for WristOx2. Valid clinical data was 63% of intended monitoring time for Lifetouch and 50% WristOx2. 29 patients experienced 36 clinically significant deteriorations. The RAPID Index detected significant deterioration more frequently (77% to 97%) and earlier than the PEW score ≥ 9/26. High sensitivity and negative predictive value for the RAPID Index was associated with low specificity and low positive predictive value. We conclude that it is feasible to collect clinically valid physiological data wirelessly for 50% of intended monitoring time. The RAPID Index identified more deterioration, before the PEW score, but has a low specificity. By using the RAPID Index with a PEW system some life-threatening events may be averted.

## Introduction

Paediatric Early Warning (PEW) systems have reduced the late or undetected clinical deterioration experienced by some hospitalised children^1–3^. Following the introduction of the bedside PEW score in Birmingham Children’s Hospital, a multidisciplinary team identified missed opportunities and recommended more continuous monitoring for visualisation of trends and a smart alarm for earlier detection of deterioration.

Continuous and intermittent monitoring has led to early identification of patient deterioration, increased rapid response activations and improvements in completeness of vital signs documentation^4,5^. Standard monitors require sensors to be hard-wired to patients and require additional software to extract data for advanced analytics^6^. Wireless monitoring offers a potentially more comfortable and economical solution although accuracy, continuity, patient tolerability and power management are challenging^7,8^. A number of sensors and monitoring systems have been developed and shown to be feasible but few have been sufficiently developed and tested in large clinical trials^7–15^. The advantage of more continuous monitoring for ward patients is that trends and Big Data analytics can be used to improve detection of deterioration^16–18^. The intent is not that smart alarms replace clinical surveillance but that they are used in addition to current systems to support and augment decision making^19–21^.

Neither continuous wireless monitoring nor integrated smart alarms have been reported in the context of paediatric wards. This study tests the feasibility of collecting sufficiently useful continuous wireless monitoring and the feasibility of a real-time smart alarm to assess whether the technology is at a clinically acceptable level. This report provides the study recruitment, wireless monitoring duration and identification of significant clinical deterioration in comparison to the PEW score.

## Methods

### Study setting and population

The study was conducted in Birmingham Children’s Hospital, a Specialist secondary, tertiary and quaternary paediatric hospital in two wards of 36 beds from November 2015 to March 2018. The patients in this study had cyanotic and acyanotic cardiac and non-cardiac conditions and were admitted for elective procedures and emergency care. The levels of care provided in this area are intermittent monitoring, continuous monitoring, continuous humidified high flow oxygen (HHFO), continuous nasal positive airway pressure (CPAP), infusions of vaso-active medications (Prostaglandin, Dobutamine and Milrinone) and internal or external pace-maker support. Patients requiring urgent Paediatric Intensive Care Unit (PICU) admissions are frequently at a high level of support (level two paediatric critical care)^22^.

### Study design

A prospective cohort study design with analysis protocol was developed before data collection commenced. South West Research Ethics Committee approval was obtained (SWREC 13/SW/0001) for children to wear two additional wireless sensors. Research nurses approached children and young people and their parents or legal guardian on the study wards to gain prospective informed consent. All methods were carried out in accordance with relevant ethical and data regulation guidance.

### Eligibility criteria

All children, from birth to 17 years, with or without a primary cardiac diagnosis, were eligible except those requiring pacemakers and those in whom informed consent could not be achieved.

### Data transmission

The WIFI in the ward was confirmed to be adequate and a medical device bandwidth was applied. The CE marked Patient Status Engine System (Isansys, Oxford, UK) was established for 36 beds in two wards. This system included a gateway (modified Samsung tablet) at each bed space and a data server behind the secure hospital firewall. The sensors and gateway were registered to a specific patient. The bedside gateways had a simple graphic display to confirm that data was being collected. A dashboard was developed to view high and low level patient data using the familiar PEW system chart. Two CE marked wireless sensors were used. The LifeTouch (Isansys, Oxford, UK) measured ECG-derived heart rate (HR) and respiratory rate (RR). The WristOx2 (Nonin, Plymouth, MN) measured pulse oximetry (SpO_2_) and derived pulse rate (PR). Data was transferred each minute by low power blue tooth to the gateways. Sensor position was checked to confirm correct placement by streaming waveforms.

### Data collection

Data was prospectively collected on patient recruitment, demographics, and clinical outcomes. The quantity, quality and accuracy of physiological data were measured throughout the study. To calculate valid clinical data we applied broad filters for each parameter to exclude technical noise. Data was excluded if values were outside the ranges of: HR < 20/min or HR > 300/min, RR < 5/min or RR > 200/min and SpO_2_ < 30%. In the absence of a standard for wireless monitoring we chose duration of intended monitoring time with clinically relevant data. We chose the usual 24 h and a longer exploratory 72 h window for identification of deterioration.

### PEW score

Our PEW system combines education, an aggregated PEW score, monitoring standards, patient specific thresholds and clinical judgement^23^. Nurses measure observations and the PEW score every one to four hours and follow guidance on routine and enhanced monitoring. The PEW score includes seven parameters: respiratory rate, respiratory distress, pulse-oximetry, inspired oxygen, heart rate, systolic blood pressure and capillary refill time^1,3^. A PEW score of ≥ 9 of 26 or significant concern triggers a request for PICU review. They can request assistance from the patient’s medical team, a nurse-led Patient at Risk Team (PART), a senior PICU clinician or the cardiac arrest team. The threshold of PEW score ≥ 9 was used for comparison with the RAPID Index.

### RAPID index

The RAPID Index was developed using bespoke machine learning techniques designed for this study. It is a novel patient-adaptive ‘index of surprise’, representing a digital equivalent of the PEW score. To develop the RAPID Index a sample of 410 training monitoring sessions of more than 4 h duration was selected between May 2015 and March 2016. Sessions of patients who had experienced clinical deterioration and sessions with only spurious data were excluded. The algorithm was trained, tested and used on raw, noisy and unfiltered data so that it can work in real-time with imperfect real-life data. The training sample was used to identify patient “phenotypes”. These phenotypes are groups of patients whose HR, RR and SpO_2_ interact in a similar way and provide an expected range against which each patient can be compared; similar to the normal range used in PEW scores. When patients deteriorate they move away from the cluster of phenotypes representing conventional behaviour. The RAPID Index provides an instantaneous measure of deviation between the probability distributions of the actual observed patient physiological interaction data and the expected patient dynamics based on their recent physiological data. In summary, the algorithmic method involved stages of: signal conditioning and feature extraction, time series modelling, dissimilarity characterisation, machine learning interpolation, data visualisation, and the calculation of the RAPID index. Signal conditioning and feature extraction involved data verification and the creation of windows of time series segments across all sensors and the use of singular vectors to eliminate unphysical high frequency events. Multiple predictive linear multivariate time series models were created to model the feature space time series vectors, and the residuals across a temporal interval were used to characterise the validity of the set of models. A measure of dissimilarity was used between time series models and data to infer how distinct each patient’s biopattern was. These patient models were visualised in a two-dimensional projection by a Neuroscale machine learning approach using the model dissimilarities to characterise the population response as represented through their sensor data. New behaviour could be observed in this distribution of the patient models of the selected population as trajectories which project into novel or sparse regions in this visualisation space. This indicated new patient behaviour and was used to define patient “phenotypes”; i.e. characteristics of patients sufficiently distinct to the existing observed population characteristics to warrant the creation of a new “patient behaviour type” we interpreted as a phenotype. So phenotypes span a medical characterisation space, representative of the patient population under consideration. The trained Neuroscale model creates a visualisation space, the geometry of which is informative of the training patient cohort. In particular the local curvature of the visualisation space indicates the data disparity. When new patients are presented to the trained architecture as described above, if their response behaviour of their sensor data is consistent with previously observed patient data, as captured in the visualisation space, then those new patients are well-represented by the existing phenotypes. A measure of their instantaneous medical “state” is determined by the local curvature of where their data projects in the visualisation space. This measure of local curvature is used as a “surprise” index, since it reflects how unusual (or not) a specific patient’s sensor responses are relative to the historic training data across many hundreds of patients. This is how the RAPID index is calculated. Whilst the patient data projections are consistent with the historic behaviour of the training data phenotypes, their RAPID index also remains consistent with the training data. However, if new patient data is projected into novel regions, the curvature changes and the RAPID index increases. The rise in the RAPID index was used in this study as a measure of patient-specific stress, and an empirically-determined threshold used to create an alarm for the nurses.

Observation of the training patient data enabled the selection of a suitable threshold value of the RAPID index to detect deterioration. The RAPID Index can be visualised as a frequency-time plot or mapped over the cluster of phenotypes in real-time within 5 min of monitoring commencing. If the deviation from the expected stable trajectory is severe the RAPID index will be higher than the threshold and an alert via the dashboard could recommend clinical review. The RAPID Index continues to monitor patients when one of the sensors temporarily ceased to transmit valid data. The missing data can be imputed using signals from the patient phenotype space to provide realistic continuous signals for 15 min.

### Reference standard—significant clinical deterioration

The target outcome condition was significant clinical deterioration which included cardiac arrest (defined as any cardiac massage), respiratory arrest (defined as needing ventilator support), other life-threatening events on the ward and unplanned PICU admissions. Two research clinicians (HD, BF) reviewed all cases with significant deterioration and reached agreement in all cases. The *onset* of deterioration was defined as escalated therapy on the ward including starting antibiotics, starting respiratory support or increasing from a stable background of support, addition of vaso-active and anti-arrhythmia medications.

### Statistical analysis

Descriptive statistics were used to describe the overall cohort and consider the feasibility of wireless monitoring. In the absence of data to inform the sample size 1,200 sessions were chosen (20% of the anticipated 6,000 admissions).

To compare the RAPID Index and a PEW score ≥ 9 the sessions with significant clinical deterioration were compared with an equal number of randomly selected control patients. For cases all alerts were considered to be true positives and those without alerts were considered to be false negatives. For controls all alerts were considered to be false positives and those without alerts were considered to be true negatives. The frequency of measurement of the PEW score is every one to four hours and the frequency of the RAPID Index is every minute.

Basic estimates of test accuracy [sensitivity, specificity, positive predictive value (PPV) and negative predictive value (NPV)] were estimated for both the RAPID index, and PEW score, along with corresponding confidence intervals. The difference in sensitivity and specificity was calculated using McNemar’s test. Estimates of PPV and NPV were calculated assuming the true prevalence was 5.6%, as seen overall in the RAPID study. Confidence intervals for the PPV and NPV estimates were calculated using the method of Mercaldo^24^.

## Results

### Population demographics

**Figure 1:**
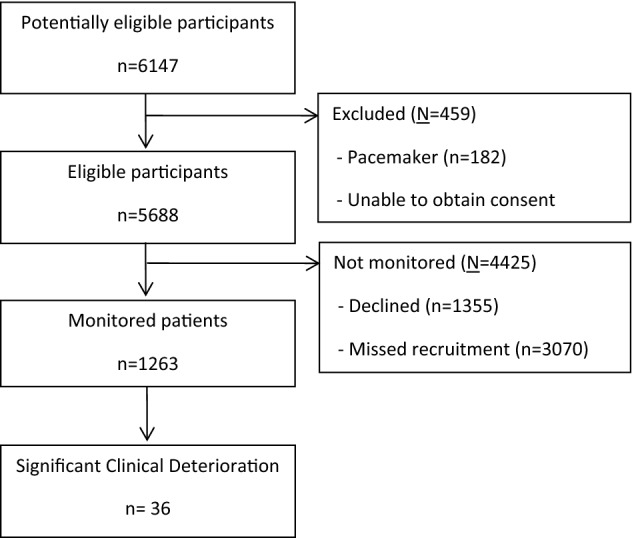
Flow diagram of recruitment and significant deteriorations.

A total of 982 patients contributed 1,263 monitoring sessions including 7,073,486 min of monitoring time (Fig. 1). Despite recruiting to the 1,200 session target only 20.5% of the total population were recruited (Table 1). The demographics of the recruited and not recruited patients were similar.

**Table 1:** Patient demographics for RAPID monitoring and whole ward population.

	**Total RAPID study sessions**	**Total ward admissions**
n =	1,263 sessions	6,147 patients
Age (months at admission); median [Q1, Q3]	27.2 [4.4, 88.8]	29.5 [4.8, 94.8]
Neonates (0–28 days at recruitment); n (%)	108 (8.6)	755 (12.1)
Male; n (%)	694 (54.9)	3,135 (51.0)
PIM3 at PICU admission; median [Q1, Q3]	0.04 [0.02, 0.07]	0.05 [0.02, 0.10]
Ward LOS (hours); median [Q1, Q3]	71 [30, 163]	51.6 [27.2, 190.6]

### Feasibility of wireless physiological monitoring

The proportion of session time recorded and validity of data (within clinical ranges) improved during the study. Modifications to software and hardware improved *data capture* from 74 to 93%. Lifetouch improved from 66 to 93% and WristOx2 improved from 49 to 55% as a proportion of intended monitoring time. *Valid clinical data* also improved: Lifetouch increased from 58 to 63% and WristOx2 increased from 23 to 50% as a proportion of intended monitoring time. Overall valid wireless data was recorded for more than 50% intended monitoring time.

### Clinical outcomes

Twenty nine patients had 36 significant deteriorations: two patients had three events and three patients had two events; 16/29 (55%) were male. The age range was four days to 16 years and the median (IQR) age was 11 (2 to 18) months. All patients with significant clinical deteriorations had an underlying primary cardiac diagnosis. The cause of the acute deterioration was heart failure or structural heart disease (n = 20), viral respiratory infection (n = 8), arrhythmia (n = 4) or gastro-intestinal disease (n = 4). The mean time from identification of deterioration to significant clinical outcome was 40.2 h for the PEW score and 46.9 h for the RAPID Index (Table 2).

**Table 2:** Time in hours from first detection of deterioration to significant deterioration or Paediatric Intensive Care admission.

**Time (hours)**	**PEW score ≥ 9**	**RAPID index**
n	31	35
Mean	40.2	46.9
Min	0.0	0.0
IQ1	8.4	23.2
Median	44.9	49.0
IQ3	64.6	66.9
Max	77.2	196.4

The onset of deterioration was identified within 72 h by the PEW score in 31/36 (86%) and RAPID Index in 35/36 (97%) of cases. RAPID Index identified patients on average 52 h earlier than the PEW score one patient was not identified by the RAPID Index (Table 3). The RAPID Index produced more alerts/ hour for cases and controls (Table 4).

**Table 3:** Sensitivity for PEW score, PEW system and RAPID Index.

**Time**	**PEW score ≥ 9**	**RAPID index**
n = 36		
Identified in 24 h; n	29	28
Not identified in 24 h; n	7	8
Identified (sensitivity) in 24 h; % (95%CI)	80.6 (64.0, 91.8)	77.8 (60.8, 89.9)
Not identified in 24 h; % (95%CI)	19.4 (8.2, 36.0)	22.2 (10.1, 39.2)
Identified in 72 h; n	31	35
Not identified in 72 h; n	5	1
Identified (sensitivity) in 72 h; % (95%CI)	86.1 (70.5, 95.3)	97.2 (85.5, 99.9)
Not identified 72 h; % (95%CI)	13.9 (4.7, 29.5)	2.8 (0.1, 14.5)

*One sided 95% CI.

**Table 4:** Comparison of sessions with and without significant deterioration.

	**Cases n = 36**		**Controls n = 36**	
	**PEW score ≥ 9**	**RAPID index**	**PEW score ≥ 9**	**RAPID index**
Total duration in hours; n	2,382	1,491	1769	1,359
Sessions with alert; n	31	35	7	27
Total alerts; n	878	2,533	44	1505

PEW score ≥ 9 has an overall better predictive discrimination; the sensitivity of RAPID Index is high with a poor specificity. Both have value to exclude those patients who are unlikely to experience significant deterioration (Table 5).

**Table 5:** Predictive value of PEW score ≥ 9 compared to RAPID Index.

**n = 72**	**PEW score ≥ 9**	**RAPID index**
Sensitivity; % (95% CI)	86.1 (70.5, 95.3)	97.2 (85.5, 99.9)
Specificity; % (95% CI)	80.6 (64.0, 91.8)	25.0 (12.1, 42.2)
Difference in sensitivity; % (95% CI)	11.1 (-4.5, 26.7)	
Difference in specificity; % (95% CI)	55.6 (34.8, 76.3)	
Positive predictive value*; % (95% CI)	20.8 (17.2, 24.9)	7.1 (7.0, 7.3)
Negative predictive value*; % (95% CI)	99.0 (98.6, 99.3)	99.3 (95.0, 99.9)

*Calculated assuming a prevalence of 5.6%.

Clinical review identified that patients experiencing 22 of the 36 significant deterioration sessions were escalated in a timely manner and 14 patients could have benefitted from earlier review. The RAPID Index identified all 14 of these patients who could have benefitted from earlier review; the PEW score identified 13 patients with enough time to intervene (Table 6). However, in all there was less than an hour between the PART and PICU doctor attending and significant deterioration. Four individual patients (aged 3 to 28 months) with potentially avoidable respiratory and cardiac arrests had RAPID monitoring for a mean of 83 h (range 20 to 218 h). The mean duration of warning was adequate to intervene; 33.4 h for the PEW score and 37.8 h for the RAPID Index. Had the wireless monitoring and RAPID Index been reviewed the four arrests could potentially have been avoided. Wireless monitoring trends were visible at the bedside but the RAPID Index smart alarm was not. We received no verbal or email feedback that indicated that clinicians used research data in real-time.

**Table 6:** Fourteen patients could have benefitted from earlier intervention.

**n = 14**	**PEW score** ≥ **9 (hours)**	**RAPID index (hours)**
Total	454.9	593.90
Mean	32.5	42.42
Median	35.8	49.2
Min	0.0	6.48
IQR 1	4.9	15.92
IQR 3	54.7	66.48
Max	72.0	71.95
No warning	1	0

## Discussion

We have demonstrated the feasibility of wireless monitoring and displaying a smart alarm in real-time in 982 hospitalised children. Depending on the sensor it is feasible to collect valid clinical data wirelessly for 50–63% of the intended monitoring time. The continuous data contributed to a novel machine-learning smart alarm, the RAPID Index that has high sensitivity, poor specificity and an earlier detection of deterioration. The PEW score has an overall better predictive discrimination, however, RAPID Index identified more (77 to 97%) significant clinical deteriorations than the PEW score.

Data loss from sensors, even when the collection system (Bluetooth, gateways, WiFi, and server) is working, is well described and less with non-mobile patients^25–28^. Patients remove the sensors, the sensors lose power, disconnect, and there is signal noise from movement between the patient and sensor. There are currently no standards for wireless data quantity, quality and spread. Previous studies report 6 to 80% loss with various devices^25–28^. The difference potentially relates to whether clinicians are using the information from these sensors and so ensuring good sensor positioning. Current routine hourly observations provide a single measure per hour. In the absence of guidance and the technology untested at this scale we chose to strive for 50% of intended monitoring time which was able to detect deterioration. The optimal sample frequency, or spread, is also not clear. In our PICU study the prediction of cardiac arrest sampling less than every 15 min had a detection advantage^29^. Sensor usability was different between the two sensors as reflected in the reduced data from the SpO_2_ sensor indicates that a better and more tolerable device is required. Although new sensors will improve the continuity and quality of data it is anticipated that wireless data will still be at least briefly discontinuous and often noisy.

Big data and smart analytics have been used with patients hard-wired to monitors with an indication that they can identify significant deterioration^17,30–32^. The RAPID Index is a new smart alarm that identifies patient-specific normality and when a patient’s physiology is deviating from the expected. Potential benefits of the RAPID Index are patient-specific earlier identification, the ability to detect deterioration when dealing with imperfect, noisy, real-time data and when only a single sensor remains attached.

Sensitivity and specificity are used to establish effectiveness of early warning scores. For the smart alarm we followed this approach although it is more relevant to a binary clinical test rather than an evolving clinical picture with interventions.

Clinical deterioration occurs on a spectrum. We defined the *onset* of deterioration as escalation of respiratory support and specific medication. Alternatives would have been to use the time that PICU or the PART was called to review the patient but this was sometimes planned or inconsistent. Previous early warning studies have used a one to 24 h windows for detection^1,3,29^. Being an observational feasibility study we chose to extend the window of detection to 72 h to identify the spectrum of deterioration, responses and outcomes. The onset of deterioration was detected by the RAPID Index about 47 h before and PEW score 40 h before the event. The long duration of warning is illuminating but does allow for more false alarms^33^. Detection was associated with further escalation of therapy until PICU was required. For early warning this means that there could be multiple cycles of alert and interventions in the period between the onset and significant clinical deterioration. The PEW score was measured every hour and the RAPID Index each minute. Therefore the test favoured the less frequent PEW score and penalised the RAPID Index and partly explains the high sensitivity and frequent false alarms. Considering the deterioration continuum and frequent sampling we need more appropriate analysis tools than sensitivity and specificity for technology assessments.

In this design, escalation of patients for medical review was based on the PEW system. The performance of the PEW score to detect deterioration in this study is similar to that previously reported but clinicians often aren’t escalating at this threshold^2,3,34,35^. This shows a human factors based over-ride that has important implications for technology developments in this area; we need to understand the ‘implicit rules’ that clinicians are following in order to maximise the potential benefit of early detection^35–38^. Paying attention to visualisation, using a recommending system with automated alerts in conjunction with advanced analytics are possible solutions^6,30,35–42^.

There are many limitations of this study mainly relating to generalisability. It was conducted in a single centre that delivers enhanced care on paediatric wards to a specific, mainly cardiac, population. Our threshold for significant deterioration may differ from institutions where higher dependency therapies are only provided in PICU. Only 20% of the ward population was recruited; similar to other studies^11,25^ although the demographics show our sample was representative of the whole population. Future efficacy studies need to offer a different model of consent in order to achieve the majority of the population as the sample^43–45^. The RAPID Index training sample for the phenotypes and algorithm identified children with mainly cardiac conditions. Paradoxically we proposed that if RAPID could work with highly variable physiology could make it more transferable to less varied populations; a theory yet to be tested. The definition and onset of deterioration was determined through consensus between two of the investigators potentially introducing bias and overfitting. In this feasibility study we did not separate out the contribution of better trend information with wireless monitoring from the RAPID smart alarm to detecting deterioration.

There are many challenges to implementing wireless monitoring and smart alarms^45^. Research into sensor and trial design, implementation, taxonomy, sample frequency, standards and analysis is required to evolve and tame the technology^45,47^. Large clinical trials with varied populations are required to establish efficacy and cost-effectiveness.

## Conclusions

Wireless monitoring in children can collect clinically valid data for more than 50% of intended monitoring time. An improved pulse oximeter sensor is required to improve data capture. We have shown it is feasible to develop and display a smart alarm in real-time. The RAPID Index can detect the majority of significant deteriorations before the current early warning system but has a low specificity and is currently associated with false alarms. The RAPID Index and PEW score are effective at ruling out the likelihood of significant deterioration. Further improvements in data quality will hopefully improve the precision. By using the RAPID Index in addition to PEW system it is possible that some life-threatening events may be averted. This data is very preliminary and the technology needs further efficacy and health economic evaluation prior to adoption.
